# Surgery versus Botulinum Toxin A for Treatment of Post‐Stroke Upper‐Limb Spasticity: White Matter Changes, Function, and Quality‐of‐Life—A Randomized Controlled Trial

**DOI:** 10.1155/np/6479640

**Published:** 2026-10-07

**Authors:** Patricia Hurtado-Olmo, Pedro Hernández-Cortés, Ángela González-Santos, Clarisa Simón-Pérez, Laura Del Olmo-Iruela, Andrés Catena

**Affiliations:** ^1^ Hand and Upper Limb Surgery Unit, Orthopedic Surgery Department, San Cecilio University Hospital of Granada, Avenue del Conocimiento s/n, Granada 18007, Spain; ^2^ Surgery Department School of Medicine Granada University Avenue Doctor Jesús Candel Fábregas 11 Granada 18016 Spain ugr.es; ^3^ Instituto de Investigación Biosanitaria IBS. Granada Avenue de Madrid, 15 Granada 18012 Spain; ^4^ Department of Physical Therapy Faculty of Health Science University of Granada Avenue de la Ilustración, 60 Granada 18071 Spain ugr.es; ^5^ BIO277 Group. A02-Cuídate Instituto de Investigación Biosanitaria IBS. Granada Granada Spain; ^6^ Hand Surgery Unit Orthopedic Surgery Department University Hospital of Valladolid Avenue Ramón y Cajal, 3 Valladolid 47003 Spain; ^7^ Rehabilitation Department, San Cecilio University Hospital of Granada, Avenue del Conocimiento s/n, Granada 18007, Spain; ^8^ Faculty of Psychology University of Granada Campus Universitario Cartuja Granada 18011 Spain ugr.es

**Keywords:** magnetic resonance imaging, neuronal plasticity, spastic upper limb, stroke, surgical procedures, white matter

## Abstract

**Introduction:**

A magnetic resonance image (MRI) study recently described greater improvements in gray matter volume and cortical thickness in post‐stroke patients with upper‐limb spasticity treated with surgery versus botulinum toxin‐A (BoNT‐A).

**Objective:**

To compare changes in the white matter of these patients after surgical versus BoNT‐A treatment and to relate findings to upper‐limb function and quality‐of‐life.

**Methods:**

In a two‐arm randomized controlled clinical trial, 30 post‐stroke patients (>12 months) with spastic upper limbs were recruited and randomly assigned (1:1) to surgery or BoNT‐A treatment (controls). Functional and quality‐of‐life parameters were studied at baseline (pre‐intervention) and at 6 months, while brain MRI scans of the white matter were taken at the same time points using a Siemens 3T Prisma MRI scanner. Although baseline MRI scans were obtained in all 30 participants, complete longitudinal MRI data were available for only 14 participants because of contraindications or refusal of follow‐up imaging. Therefore, the imaging analyses were considered only exploratory and hypothesis‐generating.

**Results:**

Increased post‐ versus pre‐surgery fractional anisotropy (FA) values were associated with Carer Burden and House functional scale scores, and decreased FA values with Ashworth, Fugl‐Meyer, pain visual analogue scale, and SF‐36 scores. Reduced mean diffusivity (MD) values for inferior fronto‐occipital fasciculus, cingulum parahippocampal parietal, uncinate fasciculus, and reticular tract were associated with improvements in Fugl‐Meyer motor recovery scores.

**Conclusion:**

The post–pre increase in FA of white matter tracts in the experimental versus control group may suggest differences in white matter microstructure between treatment groups, potentially reflecting neuroplastic changes. These findings suggest that changes in white matter microstructure might be associated with improvements in motor function, independence, and caregiver burden. However, given the small longitudinal MRI sample, the tract‐specific findings should be considered hypothesis‐generating and require confirmation in larger studies.

## 1. Introduction

The World Stroke Organization (WSO) Global Stroke Fact Sheet 2022 describes stroke as the third‐leading cause of death and disability worldwide, as expressed by disability‐adjusted life‐years lost (DALYs) [1]. More than one‐third of acute stroke survivors develop spasticity and frequently require lifelong medical treatment and the assistance of others to carry out daily life activities [2].

Post‐stroke improvements in brain structure and functions are mainly attributed to neuroplasticity mechanisms [3], with the brain being better prepared for recovery during the first few weeks and then reaching a plateau between 3 and 6 months after the stroke. Subsequently, spontaneous recovery is at its limit, and deficits usually become chronic [4], regardless of the application of multiple novel therapies.

As a complement to clinical evaluations, neuroimaging studies have found associations between post‐stroke changes in the structure of white matter and motor recovery, especially in the upper limb [5, 6], and authors have proposed their possible usefulness to assess the response to treatment [7–9].

Neuronal injury is followed by Wallerian nerve fiber degeneration, which is frequently observed in the corticospinal tract (CST) after a stroke. These alterations can be captured by means of diffusion tensor imaging (DTI), and the fractional anisotropy (FA) of white matter tracts has been found to decrease with more severe injury [10, 11]. Mean diffusivity (MD) in stroke has a characteristic time course and therefore acts as a critical biomarker for identifying tissue at different stages of ischemia. In this way, MD sharply decreases (restricted diffusion) in the acute stage due to cytotoxic edema, while it increases in subacute and chronic stages [12]. Given the capacity for activity‐dependent plasticity of white matter in the adult brain [7], these changes may be potentially useful for the clinical evaluation of post‐stroke motor recovery and prediction of the response to rehabilitation therapy [13].

The current approach to the treatment of post‐stroke upper‐limb spasticity is multidisciplinary, with intramuscular botulinum toxin‐A (BoNT‐A) injection playing a leading role [14]. This treatment has rapidly displaced surgery since its approval in the 1990s [15], despite studies that found surgical treatment to be effective and safe but under‐utilized and rarely included in clinical care protocols for post‐stroke patients [16]. Some publications have associated BoNT‐A injections with changes in the microstructure of white matter [7] but not in post‐stroke patients. Currently, there is a lack of comparative evidence on the differential effects of surgical treatment versus BoNT‐A on the white matter microstructure in patients with chronic post‐stroke spasticity, particularly in relation to clinically meaningful outcomes (e.g., motor function, quality‐of‐life, and caregiver burden). Understanding these differences may help elucidate the central mechanisms underlying treatment response and guide therapeutic decision‐making.

The aim of this longitudinal study was to explore alterations in white matter microstructure in patients with chronic post‐stroke upper‐limb spasticity using diffusion‐based magnetic resonance image (MRI) techniques. Post‐treatment changes were compared between patients who underwent surgery or repeated BoNT‐A injections, examining their association with upper‐limb performance and quality‐of‐life. We hypothesized that surgery would be associated with greater increases in FA and greater decreases in MD in sensorimotor and associative white matter tracts compared with BoNT‐A treatment because of its longer‐lasting effects on limb biomechanics and function and that these changes would be associated with improvements in motor function, quality‐of‐life, and caregiver burden.

## 2. Materials and Methods

### 2.1. Study Design

This randomized controlled clinical trial, with two parallel arms, was conducted in patients with chronic post‐stroke upper‐limb spasticity. Participants were allocated in a 1:1 ratio to receive standard treatment with botulinum toxin A (BoNT‐A; control group) or surgery (experimental group). Brain MRI scans were obtained for all participants at study enrollment and repeated at 6 months after the intervention.

The trial was registered at ClinicalTrials.gov (identifier: NCT06392633; registration date: April 30, 2024) and forms part of a broader research project (PI20/01574) [17] that previously reported on gray matter changes in a complementary analysis [18]. The trial was registered after the start of recruitment for purely administrative reasons. No changes were made to the study design, eligibility criteria, interventions, or outcomes after study initiation.

### 2.2. Participants

The sample size was estimated assuming a type I error rate of 5%, statistical power of 80%, and large expected effect size (0.8 standard deviations). Eligibility criteria included age over 18 years, diagnosis of upper‐limb spasticity secondary to stroke, a minimum post‐stroke interval of 12 months, agreement to surgical treatment when applicable, and provision of written informed consent by the patient or legal representative. Exclusion criteria were high anesthetic risk (American Society of Anesthesiologists grade ≥ IV), presence of involuntary extrapyramidal movements, inability to reliably complete questionnaires, deformities not amenable to surgical correction, or inability to ensure a minimum follow‐up of 12 months. Participants were recruited between January 2023 and December 2024, and the trial proceeded without interruption. The flow of participants is presented in Figure 1 in accordance with CONSORT guidelines [19].

**Figure 1:**
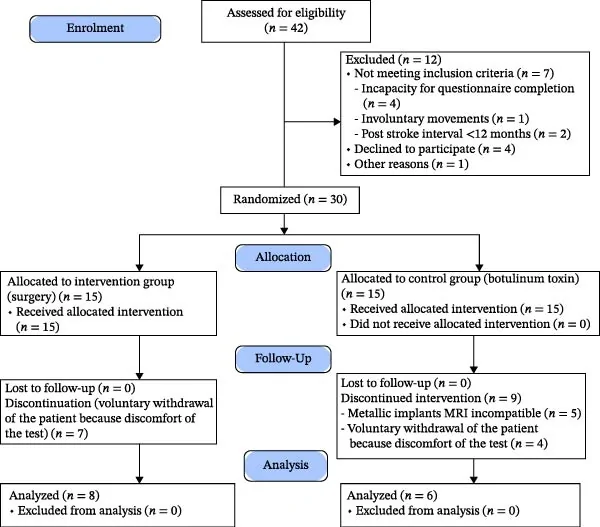
Study flowchart. CONSORT diagram.

All candidates underwent an initial clinical evaluation by an experienced rehabilitation physician at San Cecilio University Hospital of Granada, including a neurological examination and a standardized clinical interview. Eligible participants were subsequently studied by MRI and evaluated by a specialist in hand and upper‐limb surgery. Surgical candidates received an individualized operative plan for single‐session surgery that combined soft‐tissue, neural, and skeletal procedures after examination under selective peripheral nerve block anesthesia.

### 2.3. Randomization and Group Assignments

Following completion of enrollment (*n* = 30), participants were randomly assigned to either the surgical or control group. Randomization was performed by an independent investigator not involved in data acquisition using a computer‐generated allocation sequence to minimize selection bias.

Patients and the surgeon were inevitably aware of the therapy received in each case (surgery vs. BoNT‐A injection); however, the independent researchers responsible for functional clinical assessments and MRI analyses were blinded to the treatment assignment.

#### 2.3.1. Experimental Group

Participants allocated to the experimental arm (*n* = 15) were placed on the institutional surgical waiting list and underwent standard preoperative anesthetic assessment. Antiplatelet or anticoagulant therapies were temporarily replaced with low‐molecular‐weight heparin before hospital admission. Single‐event multilevel surgery was performed under appropriate anesthesia and could involve procedures at different anatomical levels, including the shoulder, elbow, forearm, wrist, and hand. Surgical techniques comprised combinations of tenotomies, tendon lengthening or transfers, selective neurectomies, and, when indicated, arthrodesis. The following protocol for surgical indications was always applied.

Spastic shoulder with passive abduction less than 75° and internal rotation contracture: indication for pectoralis major and subscapularis lengthening tenotomy. Spastic elbow with more than 100° of flexion: indication for biceps and brachialis lengthening in cases of contracture that does not improve with anesthetic block and for supraselective neurectomy if the flexion deformity improves with anesthetic block of the musculocutaneous nerve. Forearm in pronation: indication for pronator teres detachment if painful and flexor muscle lengthening is planned. Wrist: indication for superficial‐to‐profundus flexor tendon transfer for hygienic reasons and selective flexor lengthening for functional reasons, treating irreducible deformities by total wrist arthrodesis after first‐row carpectomy. Thumb deformity in the palm: indication for selective lengthening of the flexor pollicis longus and the thenar slide in cases of spasticity of the flexor pollicis brevis. Spasticity of intrinsic muscles: an indication for neurectomy of the motor branch of the ulnar nerve.

Baseline MRI scans were obtained in all patients in the experimental group, but only eight consented to the follow‐up MRI at 6 months.

#### 2.3.2. Control Group

Patients assigned to the control group (*n* = 15) received intramuscular injections of BoNT‐A targeting the spastic muscles of the upper limb. The first injection was administered after completion of the baseline clinical and MRI assessments, and a second injection was administered 4 months after enrollment. Clinical assessments and MRI acquisition at 6 months follow‐up were therefore performed approximately 2 months, or 8 weeks, after the second BoNT‐A injection, corresponding to the period of maximum therapeutic effect. All participants followed the same planned injection and assessment schedule.

The muscles injected depended on the clinical presentation in each case. They included the pectoralis major (100–150 U), teres major (100 U), latissimus dorsi (100 U), subscapularis (75 U), biceps brachii (50 U), brachialis (50 U), flexor carpi ulnaris (50–100 U), flexor carpi radialis (50–100 U), pronator teres (50 U), flexor digitorum superficialis (50–75 U), flexor digitorum profundus (50 U), opponens pollicis longus (25 U), lumbricals (50–75 U), and flexor pollicis longus (50 U). Overall, a mean of 400 U of BoNT‐A was injected into the upper extremity at each session.

Baseline MRI scans were obtained in all control patients; however, follow‐up MRI was contraindicated in five due to incompatible intracranial metallic implants and was declined by four; therefore, complete longitudinal MRI data was available for only six of the control patients.

### 2.4. Data Collection

Clinical and quality‐of‐life assessments were conducted at baseline, before administration of the assigned intervention, and at 6 months after enrollment by a rehabilitation specialist with more than 25 years of experience during a single session at the Surgery Department of the Faculty of Medicine, University of Granada. MRI acquisitions were performed at the same two time points at the Mind, Brain, and Behavior Research Center of the same institution.

### 2.5. Clinical Assessment

Baseline data included demographic variables (age, sex), stroke characteristics (type, date), time since stroke, Functional Ambulation Categories score [20], Mini‐Mental State Examination score (MMSE) [21], stroke severity measured by Rankin scale [22], Barthel Index [23], and ASA classification of anesthetic risk [24]. An unauthorized version of the Spanish MMSE was inadvertently used by the study team without permission; however, this has now been rectified with Psychological Assessment Resources (PAR) (https://www.parinc.com/). The MMSE is a copyrighted instrument and may not be used or reproduced in whole or in part, in any form or language, or by any means, without written permission of PAR. Upper‐limb involvement was categorized as functional or hygienic hand, and deformities of the elbow, wrist, and hand were classified following the criteria of Keenan et al. [25].

#### 2.5.1. Upper‐Limb Function Assessment

Upper‐limb motor performance was evaluated at both time points using the modified Ashworth scale for spasticity [26], the nine‐point house hand function scale [27], and the upper‐limb section of the Fugl‐Meyer Assessment, which includes 33 items scored on a 3‐point scale (0–2), yielding a maximum score of 66 [28].

#### 2.5.2. Hygienic/Nonfunctional Outcomes

Hygienic and nonfunctional outcomes were assessed using goal attainment scaling for hygiene, esthetics, and pain [29]; the Carer Burden Score [30] for daily care activities; and a visual analogue scale (VAS) for pain intensity [31].

#### 2.5.3. Quality‐of‐Life, Sleep, Anxiety, and Depression

The quality‐of‐life was measured using Spanish versions of the SF‐36 Health Survey [32] and the Newcastle Stroke‐Specific Quality‐of‐Life Measure [33]. Sleep quality, anxiety, and depressive symptoms were evaluated with the SATED questionnaire [34] and the Spanish‐validated Hospital Anxiety and Depression Scale [35].

### 2.6. Brain Image Acquisition and Processing

A Siemens 3T Prisma MRI scanner was used to obtain MRI scans at both time points, applying a DTI diffusion scheme to acquire 90 diffusion sampling directions with a *b*‐value of 1000 s/mm^2^, an in‐plane resolution of 1.8 mm, and a slice thickness of 5.2 mm. This slice thickness was purposefully selected in a methodological trade‐off to favor diffusion signal stability and reduce motion artifacts. The aim was to ensure consistent and feasible tractography analysis across a population of patients with upper‐limb motor impairments. The disadvantages of this approach include partial volume effects and a lower resolution of fine anatomical details. However, the completeness and quality of data and the comparability across participants were prioritized to ensure that clinically meaningful diffusion and tractography measures could be obtained in an acutely ill post‐operative stroke cohort with a focus on sensorimotor pathways. Reversed phase‐encoding b0 by TOPUP from the Tiny FSL package (http://github.com/frankyeh/TinyFSL of the FSL TOPUP, FMRIB, Oxford) was used with multi‐thread support to estimate the susceptibility artifact. Correction for current distortion and motion was done by means of the FSL Eddy program using the integrated interface in DSI Studio (“Chen” release) (http://dsi-studio.labsolver.org), which was used for processing and preprocessing. Diffusion data were reconstructed in the MNI space, employing q‐space diffeomorphic reconstruction [36] to obtain the spin distribution function [37], at a diffusion sampling length ratio of 1.25, with an output resolution in diffeomorphic reconstruction of 3 mm isotropic voxels. Restricted diffusion imaging was used to quantify the restricted diffusion [38], while DWI with a *b*‐value below 1750 s/mm^2^ was employed to calculate tensor metrics. The connectometry analysis considered the FA and MD values.

Study outcomes did not change after the start of the trial.

#### 2.6.1. Connectometry Analysis

The connectometry database contained post‐ and pre‐intervention brain images to generate a Brain Shift Index (BSI) of post‐intervention changes in white matter tracts for each patient, dividing the difference between post‐ and pre‐surgery scans by the corresponding pre‐image. Diffusion MRI connectometry [39] was applied to develop correlational tractography for correlating longitudinal changes in FA and MD values with the variables of interest (Ashworth, House, Fugl‐Meyer, Keenan and Carer Burden, VAS, Newcastle, and SF‐36) by means of nonparametric Spearman correlation. Between‐group differences in BSI were tested by obtaining correlations between the BSI and variables of interest and then calculating these correlations for each group. A T‐score threshold of 2.5 was assigned and tracked using a deterministic fiber tracking algorithm [40] to obtain correlational tractography, excluding the cerebellum and placing the seeding region in the whole brain. Tracks were filtered by topology‐informed pruning [41] with 16 iterations and were selected by applying a false discovery rate (FDR) threshold of 0.01. The FDR was estimated by applying 10,000 randomized permutations to the group label and obtaining the null distribution of the track length. DSI Studio computes a *p*‐value at each voxel based on the regression t‐statistic. It then applies adaptive FDR correction, which is robust to dependency [42], to control the expected proportion of false positives among significant findings. Because behavioral variables were analyzed separately (one regression per behavioral measure), FDR correction was applied in each behavioral model across all white‐matter voxels, which is consistent with the standard practice in voxel‐ or tract‐wise neuroimaging regression. Spearman correlations were then calculated between the post–pre change in FA and the post–pre change in the variables of interest, using the correlations to identify tracts with statistically significant between‐group differences and applying the Yekutieli correction for multiple comparisons [43]. This correction is a conservative FDR‐controlling procedure that guarantees FDR control under arbitrary dependence among tests; in contrast, the standard Benjamini–Hochberg method assumes independence or positive dependence.

### 2.7. Statistical Analysis

The primary outcome was the change in white matter microstructure assessed by diffusion MRI connectometry, specifically the FA and MD. The BSI, automatically computed by DSIstudio, was used as a derived metric to quantify longitudinal changes.

Secondary outcomes included the associations between connectometry parameters (FA, MD, or BSI) and clinical measures, including upper‐limb motor function, spasticity, hand function, pain, caregiver burden, and quality‐of‐life, explored to assess the clinical relevance of imaging findings.

The R mice package was used to impute missing values by the predictive mean matching (PMM) method and the default *m* ( = 5) parameter with 50 iterations, considering all other numerical variables as predictors. Values were only missing for Fugl‐Meyer in one patient, the Newcastle scale in two, and the professional workload score in one. Given this low proportion of missing data (<2% of total score) and its non‐systematic pattern among participants, we used the Multiple Imputation by Chained Equations (MICE) package in R. Ten imputed datasets (*m* = 10) were generated, and PMM was applied. The imputation models included age, sex, group, affected side, and the remaining clinical covariates as predictors, excluding identifiers (subject code and session factor) to prevent overfitting. We used the first completed dataset in subsequent analyses. The gold standard for multiple imputation involves combining estimates using Rubin’s rule to consider both within‐ and between‐imputation variance. We selected a simpler approach due to the very small number of missing values and because the objective was to develop a coherent dataset for statistical inference so that uncertainty was not a key issue. The utilization of a single imputed dataset is commonly accepted for the pragmatic handling of limited data gaps in clinical studies. In fact, we were able to confirm that the imputed values were consistent with the observed distributions by using Rubin’s rules to obtain summary statistics.

Correlation analysis was performed to detect differences in white matter tracts between pre‐ and post‐treatment, as indicated by the BSI, in relation to the pre–post changes in Ashworth, House, Keenan, Fugl‐Meyer, Carer Burden, Newcastle, and SF scores. In the case of missing data, analyses were based on the intention to treat. After calculating differences in the variables of interest between the evaluations for each study group, Fisher’s test was applied to compare the correlations between the groups. This allowed correlations between white matter tracts and predictors of interest to be examined across groups, controlling for age, sex, and the brain hemisphere involved.

## 3. Results

### 3.1. Demographics and Behavior

The study included 14 participants (5 females) with a mean age of 58.6 years (58.5 and 58.6 years, respectively, for control and experimental groups). There was no significant between‐group difference in the distribution of brain hemisphere damage (5L, 3R in the experimental group; 4L, 3R in the control group). Table 1 displays the baseline data for both groups. Table 2 exhibits BSI results for behavioral measures, showing statistically significant changes in Fugl‐Meyer and Carer Burden values but non‐significant differences in House and Newcastle scores, with a direction of change that was always in favor of the surgery group. The BSI is positive when the score is higher after than before the intervention and negative when it is higher before than after. Cohen’s *q* scores were computed as a measure of the difference in association strength, finding the minimum Cohen’s *q* to be 2.29 for Table 2 and 1.98 for Table 3; these are very large effect sizes, given that a score >0.50 is considered to indicate a large effect.

**Table 1:** Baseline demographic/clinical data by treatment group.

Group	Sex (male/female)	Age (years)	Side		Type	Years since stroke	Carer Burden	Stroke severity		House	Ashworth	Fugl‐Meyer	VAS	SF‐36
			Right/left		Ischemic/hemorragic			Rankin scale	Barthel index					
Toxin	4/2	60.83 ± 10.62	2/4		4/2	4.80 ± 1.78	7.16 ± 5.49	3.33 + 0.21	68.33 ± 8.13	2.00 ± 2.44	4.33 ± 1.21	61.40 ± 28.76	4.83 ± 1.72	36.25 ± 33.50
Surgery	5/3	58.62 ± 13.63	3/5		7/1	4.14 ± 2.79	7.75 ± 3.24	3 ±0,26	73.12 ± 5.08	1.5 ± 0.75	5.00 ± 1.06	43.37 ± 19.33	4.00 ± 2.32	47.50 ± 20.87
Test	Chi‐square	Mann–Whitney	Chi^2^		Chi^2^	Mann–Whitney	Mann–Whitney	Mann–Whitney	Mann–Whitney	Mann–Whitney	Mann–Whitney	Mann–Whitney	Mann–Whitney	Mann–Whitney
*p*‐Value	0.657	0.604	0.28		0.33	0.317	0.755	0.384	0.601	0.571	0.238	0.306	0.239	0.495
95% IC (ll/UL)	−0.396/0.439	−10.9/11.0	—		−2.0/3.0	−8.0/3.0	—	—	−1.0/5.0	−3.0/0.1	−6.0/46.0	−2.0/4.0	−49.0/11.6	

*Note:* Data represented as means ± standard deviation. Confidence intervals were computed using bootstrap approach with 5000 random samples. Carer Burden: Caregiver burden scale; House: Functional scale of the hand; Ashworth: Spasticity intensity scale; Fugl‐Meyer: Neurologic functional scale; VAS: Pain Visual Analog Scale; SF‐36 quality‐of‐life questionnaire, S1 section general health.

**Table 2:** Summary statistics for the Brain Shift Index in the control and surgery groups and *p*‐value of between‐group differences.

Group	Control (*n* = 6)	Surgery (*n* = 8)	*p*	95% CI LL	95% CI UL	*p*‐Control	*p*‐Surgery
Ashworth	−0.33 (0.21)	−0.57 (0.08)	0.344	−0.31	0.78	0.18	0.001
Fugl‐Meyer	−0.04 (0.09)	0.53 (0.12)	0.01 ^∗^	−0.88	−0.24	0.69	0.003
House	−0.05 (0.05)	0.75 (0.3)	0.06^a^	−1.51	−0.09	0.36	0.04
Keenan	−0.27 (0.17)	−0.58 (0.13)	0.186	−0.16	0.78	0.18	0.003
Carer Burden	0.12 (0.41)	−0.85 (0.12)	0.04 ^∗^	−0.07	2.02	0.78	0.003
VAS	−0.06 (0.06)	−0.32 (0.13)	0.146	−0.05	0.59	0.36	0.04
Newcastle	−0.03 (0.06)	−0.18 (0.04)	0.06^a^	−0.02	0.33	0.71	0.004
SF36	0.03 (0.10)	0.03 (0.17)	0.98	−0.45	0.43	0.82	0.846

Abbreviations: BSI, Brain Shift Index; SEM, Standard error of the mean; VAS, Visual Analog Scale.

^∗^Statistically significant between‐group difference (*p* < 0.05).

^a^Trend towards significance.

**Table 3:** Between‐group differences in the association of increased fractional anisotropy (FA) in white matter tracts with the variables of interest.

Variable	Tract	H	*t*‐Control (*n* = 6)	*t*‐Surgery (*n* = 8)	*p*‐Value	95% LL	95% UL
Carer Burden	Corticostriatal tract posterior	L	−0.990	0.974	0.0000	−6.23	−3.37
	Middle longitudinal fasciculus	R	−0.996	0.867	0.0000	−5.86	−3.00
	Corticospinal tract	L	−0.981	0.956	0.0000	−5.65	−2.79
	Corpus callosum tapetum	—	−0.945	0.979	0.0000	−5.48	−2.62
	Corticospinal tract	R	−0.996	0.585	0.0000	−5.23	−2.37
	Medial lemniscus	R	−0.987	0.664	0.0002	−4.73	−1.87
	Corpus callosum forceps minor	—	−0.936	0.907	0.0003	−4.65	−1.78
	Corticopontine tract parietal	L	−0.810	0.964	0.0005	−4.55	−1.69
	Uncinate fasciculus	R	−0.936	0.876	0.0007	−4.49	−1.63
	Thalamic radiation posterior	L	−0.941	0.849	0.0009	−4.43	−1.57
	Corpus callosum forceps major	—	−0.535	0.966	0.0065	−4.06	−1.19
	Thalamic radiation superior	R	−0.913	0.774	0.0078	−4.01	−1.14
	Arcuate fasciculus	R	−0.953	0.586	0.0088	−3.96	−1.10
	Fornix	R	−0.955	0.543	0.0099	−3.93	−1.06
	Extreme capsule	R	−0.920	0.676	0.0139	−3.85	−0.98
	Inferior fronto‐occipital fasciculus	R	−0.920	0.655	0.0158	−3.81	−0.94
	Thalamic radiation superior	L	−0.393	0.955	0.0213	−3.73	−0.87

House	Reticular tract	L	−0.999	0.878	0.0000	−6.00	−3.74
	Corticopontine tract frontal	R	−0.987	0.804	0.0001	−5.04	−2.18
	Corpus callosum body	—	−0.985	0.657	0.0006	−4.67	−1.80
	Inferior fronto‐occipital fasciculus	R	0.999	0.730	0.0011	1.65	4.51
	Extreme capsule	R	0.999	0.595	0.0011	1.62	4.48
	Parietal aslant tract	L	0.998	0.424	0.0012	1.57	4.43
	Thalamic radiation anterior	R	0.998	0.655	0.0082	1.20	4.06

*Note:* H: Brain hemisphere of the tract. t: Spearman correlation for control and surgery. The *p*‐value is corrected for multiple comparisons using the Yekutieli procedure. 95% CI limits (low, LL and high, UL) are in the *z* scale.

### 3.2. Tracts

A post–pre increase in FA was associated with Carer Burden and House measures (Table 3, Figure 2A), whereas a post–pre decrease in FA was associated with Ashworth, Fugl‐Meyer, VAS, and SF‐36 values (Table 4, Figure 2B).

**Figure 2:**
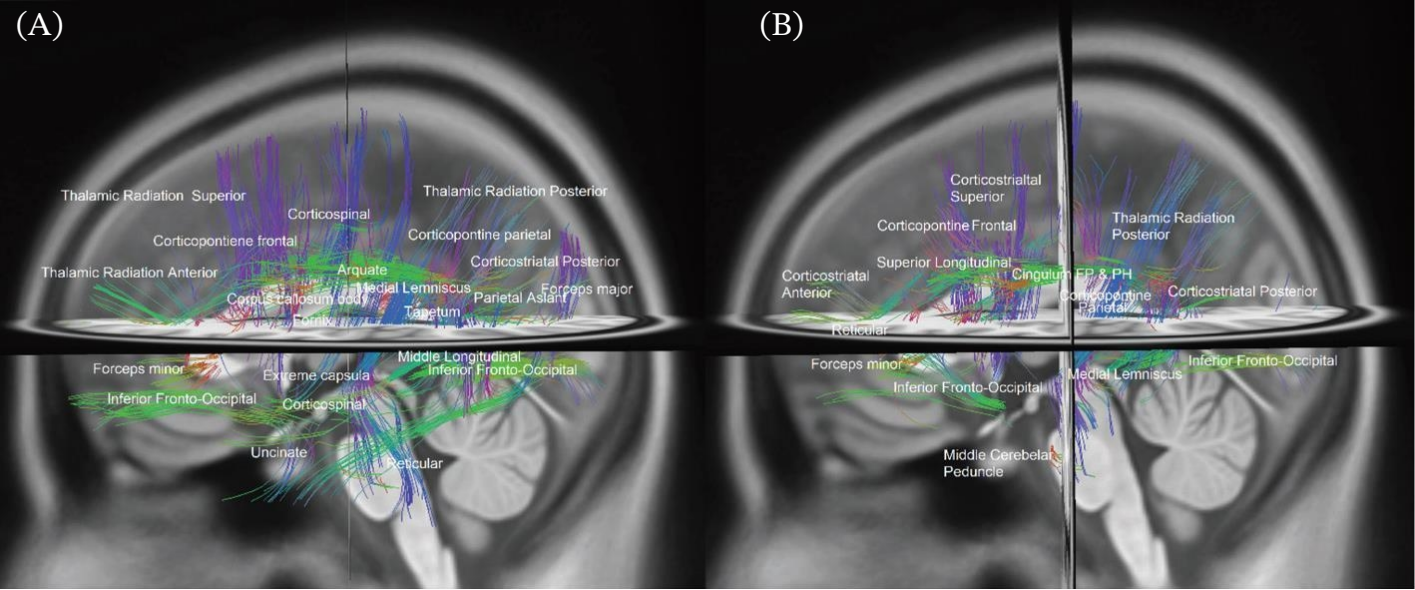
Post–pre changes in fractional anisotropy (FA) across white matter tracts. (A) Regions showing higher FA values associated with Caregiver Burden and House scores. (B) Regions showing lower FA values associated with Ashworth, Fugl‐Meyer, SF‐36, and VAS scores. Color code: Red = left–right; Green = anterior–posterior; Blue = inferior–superior (DSI Studio default).

**Table 4:** Between‐group differences in the association of a decrease of fractional anisotropy (FA) in white matter tracts with the variables of interest.

Variable	Tract	H	*r*‐Control (*n* = 6)	*r*‐Surgery (*n* = 8)	*p*‐Value	95% LL	95% UL
Ashworth	Cingulum frontal parahippocampal	R	0.997	−0.463	0.0000	2.40	5.26
Fugl‐Meyer	Corpus callosum forceps minor	—	−0.986	0.210	0.0055	−4.12	−1.26
	Corticostriatal tract posterior	L	−0.988	0.106	0.0055	−4.11	−1.25
	Corticopontine tract frontal	L	−0.963	0.383	0.0166	−3.82	−0.95
	Corticostriatal tract anterior	L	−0.961	0.319	0.0172	−3.72	−0.85
	Corticostriatal tract superior	L	−0.876	0.721	0.0172	−3.70	−0.84
	Superior longitudinal Fasciculus 2	L	−0.903	0.461	0.0495	−3.42	−0.56

SF36	Corticopontine tract parietal L	L	0.992	−0.929	0.0000	2.95	5.81
	Medial lemniscus	L	0.943	−0.959	0.0000	2.26	5.12
	Thalamic radiation posterior	L	0.968	−0.919	0.0000	2.21	5.07
	Reticular tract	R	0.999	−0.406	0.0001	2.79	5.65
	Corticostriatal tract posterior	L	0.872	−0.921	0.0009	1.51	4.37
	Middle cerebellar peduncle	—	0.873	−0.920	0.0009	1.50	4.36
	Inferior fronto‐occipital fasciculus	L	0.502	−0.959	0.0089	1.045	3.91
	Corticostriatal tract anterior	L	0.707	−0.864	0.0308	0.76	3.62

VAS	Medial lemniscus	R	0.994	−0.477	0.0001	2.00	4.87
	Superior longitudinal Fasciculus 2	R	0.682	−0.965	0.0026	1.41	4.28
	Thalamic radiation anterior	R	0.928	−0.657	0.0159	1.00	3.86

*Note:* H: brain hemisphere of the tract. t: Spearman correlation for control and surgery groups. The *p*‐value is corrected for multiple comparisons using the Yekutieli procedure. 95% CI limits (low, LL and high, UL) are in the *z* scale.

Between‐group differences in MD reduction were observed for Ashworth and Fugl‐Meyer scores (Table 5). A reduction in MD is considered to indicate improvements in water diffusion along the tracts, and both anterior thalamic radiation and anterior corticostriatal tracts are related to motor control and muscle tone. Hence, this association may be related to post‐stroke recovery. Finally, a post–pre reduction of the MD in inferior fronto‐occipital, cingulum parahippocampal parietal, uncinate fasciculus, and reticular tract is associated with Fugl‐Meyer‐assessed improvements in motor recovery, reflecting potential differences in sensorimotor, cognitive, and emotional white matter pathways. Given the small sample size, no causal inferences can be drawn from these findings.

**Table 5:** Between‐group differences in the association of decreased mean diffusivity (MD) in white matter tracts with the variables of interest.

Variable	Tract	H	*r*‐Control (*n* = 6)	*r*‐Surgery (*n* = 8)	*p*‐Value	95% LL	95%UL
Ashworth	Thalamic radiation anterior	L	−0.999	−0.462	0.000	−4.73	−1.87
	Corticostriatal tract anterior	L	−1.000	−0.905	0.001	−4.88	−2.02

Fugl‐Meyer	Inferior fronto‐occipital fasciculus	R	−1.00	0.37	0.000	−6.77	−3.91
	Cingulum parahippocampal parietal	L	−0.99	0.50	0.000	−4.63	−1.76
	Uncinate fasciculus	L	−0.94	0.83	0.004	−4.36	−1.49
	Reticular tract	L	−0.99	0.02	0.019	−4.10	−1.24

*Note:* H: the brain hemisphere of the tract. The Spearman correlation for control and surgery groups. *p*‐Value is corrected for multiple comparisons using Yekutieli. 95% CI limits (low, LL and high, UL) are in the *z* scale.

### 3.3. Safety and Adverse Effects

In the toxin group, the sole complication or adverse effect recorded was pain at the injection site. In the surgical group, there were two cases of incomplete correction of thumb deformity but no serious or systemic complications, and no wound infections were observed.

## 4. Discussion

FA is a marker of the integrity of white matter tracts [44] and its reduction may indicate structural damage, including the demyelination or disorganization of fiber tracts. Decreased FA has been observed in several brain conditions, such as mild traumatic brain injury and intraventricular hemorrhage [45], and it has also been associated with aging processes [46]. Increased FA is related to greater grip strength in stroke patients [47, 48]. In the present series, decreased anisotropy in white‐matter tracts was associated with deficits in motor, sensory, and cognitive processes. The ways in which variable scores may be influenced by post–pre FA changes in specific tracts are discussed below.

Because complete longitudinal MRI data were available for only 14 participants, including eight in the surgery group and six in the BoNT‐A group, neuroimaging findings should be interpreted as exploratory.

The Ashworth scale assesses muscle spasticity. A positive association between a post–pre decrease in FA and the Ashworth score was observed in the control group, whereas a negative association was found in the surgery group. This between‐group difference was statistically significant in the right parahippocampal cingulum, a tract potentially involved in motor control and post‐stroke brain plasticity [49]. Although this finding may reflect differences in neural mechanisms associated with muscle‐tone regulation, no causal inference can be drawn.

The Fugl‐Meyer score reflects motor recovery after stroke. A positive association between a post–pre decrease in FA and the Fugl‐Meyer score was observed in the control group, whereas a negative association was found in the surgery group. These between‐group differences involved the corpus callosum forceps minor, anterior, posterior, and superior corticostriatal tracts and superior longitudinal fasciculus II. These pathways contribute to interhemispheric communication, sensorimotor integration, and movement planning and execution [50–53]. Their involvement may therefore be related to differences in motor recovery between groups, although these exploratory associations require confirmation in larger samples.

The SF‐36 assesses the perceived quality‐of‐life across multiple domains. Associations between reduced FA and SF‐36 scores involved several sensorimotor and associative pathways, including the parietal corticopontine tract, medial lemniscus, posterior thalamic radiation, reticular tract, corticostriatal pathways, middle cerebellar peduncle, and inferior fronto‐occipital fasciculus. These tracts contribute to motor coordination, sensory processing, spatial integration, and functional performance [54–59]. Their involvement may therefore reflect the multidimensional relationship between white matter microstructure and the perceived quality‐of‐life after stroke.

The VAS is a subjective measure of perceived pain. Associations between reduced FA and VAS scores involved the medial lemniscus, anterior thalamic radiation, and superior longitudinal fasciculus II, pathways related to sensory processing and pain perception. The observed between‐group differences may therefore be associated with differences in pain‐related outcomes between surgery and BoNT‐A treatment (Table 2), although these findings remain exploratory.

A post–pre increase in FA was also observed in several tracts differentially associated with House and Carer Burden scores. For the House scale, greater increases in FA were positively associated with functional improvement in the surgery group, whereas the corresponding associations were negative in the control group. The implicated pathways, including reticular and corticopontine tracts, the corpus callosum, parietal aslant tract, inferior fronto‐occipital fasciculus, extreme capsule, and thalamic radiations, contribute to sensorimotor integration and the planning and execution of voluntary movement [60–64]. These findings may therefore reflect processes related to functional recovery and patient autonomy, although no causal inference can be drawn.

Numerous white matter tracts were also associated with Carer Burden scores. Motor pathways, including corticostriatal, corticospinal, and middle longitudinal tracts, may be particularly relevant because changes in patient mobility and functional independence could influence the amount of physical assistance required from caregivers [65, 66]. The involvement of callosal pathways may similarly reflect processes related to interhemispheric motor integration and functional dependence.

Associations with Carer Burden also involved parietal corticopontine and thalamic pathways, as well as associative and limbic tracts such as the uncinate fasciculus, arcuate fasciculus, fornix, and extreme capsule. These pathways participate in sensorimotor, cognitive, and emotional processing [67–69]. Their involvement suggests that the caregiver burden may be related not only to physical dependence but also to broader functional and psychosocial dimensions of recovery.

We previously studied structural changes in the gray matter of the same patients after toxin or surgical treatments [18]. As also observed in their white matter, neurostructural changes were primarily recorded in the surgical group rather than in the control group. In both studies, brain changes were associated not only with motor function scale scores but also with quality‐of‐life, pain, and caregiver burden. The changes were found to converge in frontolimbic, cognitive, and emotional networks and not solely in the corticospinal tract (CST). These observations suggest that the outcomes of upper‐extremity surgery may extend beyond motor function, with potential effects on emotional and social domains.

This study is limited by the small effective sample size for longitudinal MRI analyses, in part attributable to dropouts and contraindications for follow‐up imaging. This reduces its statistical power and precludes definitive causal inferences; therefore, these findings should be interpreted as exploratory and hypothesis‐generating. In addition, the selection of 5.2 mm slice thickness in the diffusion MRI study may have partial volume effects and limit the resolution of fine anatomical details; however, it prioritizes diffusion signal stability and mitigates motion artifacts, which is especially important in patients with neurological disorders.

Although participants in the BoNT‐A group were assessed according to a standardized nominal schedule, approximately 8 weeks after the second injection, individual variability in the onset, magnitude, and duration of BoNT‐A effects may have influenced muscle tone, pain, motor performance, upper‐limb use, caregiver burden, and MRI measurements. Assessment timing within the BoNT‐A treatment cycle therefore remains a potential source of bias in the comparison with the surgical group.

Finally, patients undergoing surgery no longer received repeated BoNT injections; hence, microstructural changes observed after surgery cannot be fully disentangled from changes potentially associated with the discontinuation of BoNT‐A therapy. The study strengths include the randomized design and the novelty and potential relevance of its findings.

## 5. Conclusions

An increase in post–pre FA values was observed in various white‐matter tracts after surgery, but no comparable changes were detected after BoNT‐A treatment. In the surgery group, these microstructural white matter changes co‐occurred with improvements in motor function, independence, and certain aspects of emotional/cognitive processing and with a reduction in the caregiver burden. However, given the limited sample size and the exploratory nature of the connectometry analyses, these results should be interpreted as hypothesis‐generating rather than evidence of a causal relationship. Future longitudinal studies with larger cohorts and longer follow‐ups are needed to determine whether these associations reflect a direct effect of surgery on the white matter microstructure or can be explained by other factors.

## Author Contributions

Conceptualization: Pedro Hernández‐Cortés. Methodology: Pedro Hernández‐Cortés, Ángela González‐Santos, Laura Del Olmo‐Iruela, and Andrés Catena. Formal analysis: Andrés Catena. Investigation: Patricia Hurtado‐Olmo, Pedro Hernández‐Cortés, Ángela González‐Santos, Clarisa Simón‐Pérez, and Laura Del Olmo‐Iruela. Data curation: Clarisa Simón‐Pérez. Writing – original draft: Patricia Hurtado‐Olmo, Pedro Hernández‐Cortés, and Andrés Catena. Writing – review and editing: Pedro Hernández‐Cortés, Ángela González‐Santos, Clarisa Simón‐Pérez, Laura Del Olmo‐Iruela, and Andrés Catena. Funding acquisition: Pedro Hernández‐Cortés.

## Funding

Project “PI20/01574” was funded by Instituto de Salud Carlos III (ISCIII) and co‐funded by the European Union. This research forms part of the doctoral thesis of Patricia Hurtado‐Olmo, developed under the Doctoral Program in Clinical Medicine and Public Health at the University of Granada (Spain).

## Disclosure

All authors have read and agreed to the published version of the manuscript.

## Ethics Statement

This study received approval from the clinical research ethics committee of the Junta de Andalusia (Spain) on December 7, 2020 and follows the ethical criteria of the 2013 modification of the Helsinki Declaration for the performance of research projects and national legislation on clinical trials (Law 29/2006 26 July, modified in July 2013). The confidentiality of the study material complies with national legislation on personal data protection and digital rights (Law 3/2018, 5 December).

## Conflicts of Interest

The authors declare no conflicts of interest.

## Data Availability

De‐identified sections of the dataset will be available from the time of publication.
